# Standardized terminology and visual atlas of the external morphology and terminalia for the genus *Scaptomyza* (Diptera: Drosophilidae)

**DOI:** 10.1080/19336934.2021.1969220

**Published:** 2021-10-12

**Authors:** Augusto Santos Rampasso, Patrick Michael O’Grady

**Affiliations:** Cornell University, Department of Entomology, Ithaca, USA

**Keywords:** Anatomy, descriptions, nomenclature, synonyms, taxonomy

## Abstract

The genus *Scaptomyza* is one of the two Drosophilidae genera with Hawaiian endemic species. This genus is an excellent model for biogeographic studies since it is distributed throughout the majority of continents, including continental islands, the Hawaiian Islands, and many other remote oceanic islands. This genus currently comprises 273 described species, 148 of which are endemic to the Hawaiian Islands. However, most descriptions were published before efforts to standardizing the morphological terminology across the Diptera were made in the 1980’s. Since research groups developed their own set of terminologies independently, without considering homologies, multiple terms have been used to refer to the same characters. This is especially true for the male terminalia, which have remarkable modifications within the family Drosophilidae. We reviewed the *Scaptomyza* literature, in addition to other studies across the Drosophilidae and Diptera, compiled the English synonyms, and provided a visual atlas of each body part, indicating how to recognize the morphological characters. The goal of the present study is to facilitate species identification and propose preferred terms to be adopted for future *Scaptomyza* descriptions.

## Introduction

The Hawaiian Drosophilidae is the oldest and the most diverse clade in the Hawaiian Archipelago [1], currently containing 689 described species [2], 564 of which are endemic to the Hawaiian Islands [2], and potentially hundreds of species present in collections that remain undescribed [3,4]. Phylogenetic analyses suggest the genus *Scaptomyza* is monophyletic and the sister lineage of the Hawaiian *Drosophila* [4,5]. The genus *Scaptomyza* has a remarkable biogeography, and there are two hypotheses to describe their pattern of origin and diversification. One hypothesis suggests this genus originated in Hawaii, undergone extensive diversification, and subsequently dispersed to the mainland and other islands [4–6]. An alternative hypothesis proposes *Scaptomyza* originated in the mainland and then colonized the Hawaiian Islands in at least two independent events [7]. The genus *Scaptomyza* currently comprises 273 described species [2], 148 of which are endemic to the Hawaiian Islands [4]. About 63% of *Scaptomyza* species may only be found in remote oceanic islands, such as the Hawaiian Islands, the Marquesas, Tristan da Cunha, and Saint Helena Islands [5]. The other 101 species are distributed on all continents, including continental islands such as Japan and Taiwan, except Antarctica [8,9].

The genus *Scaptomyza* was erected on mid-1800’s [10]. Early descriptions were mostly based on brief external morphological analyses, referring to broad terms such as antennae, head, thorax, wings, and abdomen, in addition to body and wing lengths [11,12]. Species descriptions have become progressively more complex over time, as some authors started including more detailed external morphology analyses in their descriptions [13,14]. The taxonomy of *Scaptomyza*, as well as the Drosophilidae as a whole, advanced further with the inclusion of male terminalia descriptions and illustrations [15], since it became clear through culturing and crossing species in lab conditions that there were cryptic species, undistinguishable by external morphology [16]. Throughout the 20^th^ century, analysis of male terminalia started occupying a central role in *Scaptomyza* taxonomy [17], and it remains the main characteristic used to define species in modern descriptions [9].

Technological advances in the last half of the 20^th^ century contributed to a greater refinement in species descriptions. For example, better stereomicroscopes made it possible to obtain higher image resolution, which allowed taxonomists to include several indexes of body regions, setae, and wings markers [18]. During the 21^st^ century, as molecular techniques became widespread, some researchers began to apply DNA sequences as tools for species delimitation. A common modern method used to define species is DNA barcoding, an approach that uses a fragment of 658 base pairs of mitochondrial DNA (mtDNA) from the cytochrome c oxidase subunit I (COI) gene [19]. More recently, it became possible to use multilocus or genomic data for species delimitation, which would also provide insights into the processes of biodiversity formation [20–22]. It is worth noting that no *Scaptomyza* species has been described using molecular approaches and that these methods have their limitations [23]. This is particularly true of single-locus analyses, since one gene alone may not provide sufficient information to describe a new species [24,25]. These molecular tools will likely continue to be one extra source of data in integrative taxonomy studies, instead of being adopted as the only source of data [26]. Therefore, the emerging molecular tools may be most useful to distinguish cryptic insect species [27] or species that present homoplasious characters [20].

**Figure 1.**
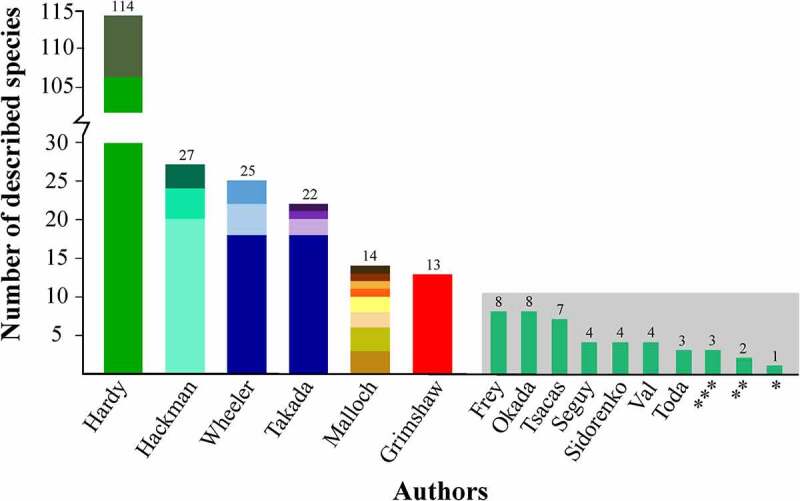
Number of described species by author. The stacked bars indicate the proportion of species described in each author’s publications, for authors that described more than 10 species [17,34–42,46,48,50,52,54,56,62,65,67,79]. The columns in the grey area correspond to authors that described fewer than 10 species, considering the total number of described species and not the number of descriptions per publication. Abbreviations: * = Bahng, Becker, Beppu, Bock, Burla, Chatterjee, Chetterjee, Collin, Coquillett, Dash, De Meijere, Goni, Grimaldi, Gupta, Kaneshiro, Kang, Knab, Kumar, Lamb, Lee, Loew, Meigen, Momma, Nishiharu, Rakshit, Singh, Thomson, and Vilela; ** = Fallen, Harrison, Lin, Ting, Walker, and Wirth; *** = Bonacum, Brncic, Cogan, DeSalle, Duda, McEvey, O’Grady, and Zetterstedt

The 273 formally known *Scaptomyza* species were described in 68 publications. However, most of those descriptions were published by independent research groups (Figure 1) before recent standardized terminology proposals were made [18,28–33]. Often, the same characters are referred to by numerous terms, making comparisons between species a difficult task, especially if drawings and/or images are not provided. This is particularly true for male terminalia characters, which exhibit unusual modifications within Drosophilidae [17].

The purpose of the present study is threefold: 1) compile different English terms used to refer to homologous characters throughout *Scaptomyza* literature, in addition to selected publications; 2) propose a standardized terminology for morphological characters used to describe *Scaptomyza*; 3) provide a visual atlas illustrating these characters to standardize across historical and future *Scaptomyza* descriptions.

## Materials and methods

### Terminology revision

The 68 papers in which all 273 formally known *Scaptomyza* species were described were examined, and the terminology adopted in the 53 English publications was compiled [9,11–14,17,31,34–79]. Since the majority of work on standardization of morphological nomenclature over the past three decades has taken place in English and this is the language used by the majority of modern *Scaptomyza* publications, we excluded 15 publications, all but three of which were written prior to WWII, written in French [80–83], German [84–90], and Latin [91–94]. The publications that erected the genus *Scaptomyza* [10], proposed new subgenera without describing new species [95,96], redescribed *Scaptomyza* species [33,97,98], described a *Scaptomyza* species that was transferred to another genus [99], proposed new combinations [100], and other selected publications relevant to modern Drosophilidae taxonomy [15,16,18,28–30,32,101–114] were also included.

### Imaging

An isofemale strain of *Scaptomyza pallida* Zetterstedt, 1847^92^, collected near Strawberry Creek on the University of California, Berkeley, campus by the Whiteman Lab, was used as our model for the visual atlas. Since individuals belonging to this species do not present secondary sexual dimorphism, both males and females were used for imaging. Therefore, the body regions pictured in the present publication do not necessarily belong to the same individual, but all flies belong to the same inbred line and to the same generation.

Flies were preserved in 70% ethanol and dissected using a pair of entomological pins. Microscope slides of antennae, legs, wings, and halteres were prepared using Euparal as mounting medium. The terminalia dissection technique is based on Wheeler & Kambysellis [115], Kaneshiro [116] and Bächli *et al*. [33]. To facilitate terminalia dissections, 22 individuals were pointed and kept at room temperature for one week to allow the exoskeleton and soft tissues to completely dry. The distal portion of the abdomen was then removed and softened by submerging in water for at least 2 hours. Dissected abdomens were transferred into a depression slide filled with water, where the terminalia was disarticulated using a pair of entomological pins. Finally, microscope slides were prepared, also using Euparal as the mounting medium.

All slides were stored at room temperature for at least one week. Slides containing antennae, legs, wings, and halteres, as well as the head and thorax of two pointed individuals, were captured at different depths of focus using an Excelis HD Microscope Camera with an 11.6-inch AU-600-HDS monitor attached to a Nikon SMZ1500 stereomicroscope, with 30×-50× magnification. The photos were stacked into an all-in-focus composite using the software CombineZP [117], according to Vilela & Goñi [118] and Vilela & Pietro [119]. Terminalia slides were imaged under a Macroscopic Solutions Macropod Pro and Canon EOS 6D DSLR camera body using EF 70–200 mm zoom lens with 50× Mitutoyo objective lens. Images were stacked using Zerene Stacking Software Version 1.04 (Zerene Systems, LLC 2014). All images were edited using Adobe Photoshop 2021 to remove the background and correct colour and white balance. Adobe Photoshop 2021 was also used to draw the line contour of the thorax in left lateral view, whereas Adobe Illustrator 2021 was used to design the bar chart.

## Results and discussion

The morphological terms adopted in the 82 analysed publications were compiled into a series of tables, according to the following major body regions: head (Tables 1 and Tables 2), antennae (Table 3), thorax (Tables 4–Tables 6), legs (Table 7), wings and halteres (Table 8), and male (Table 9) and female (Table 10) terminalia. We propose a standardized terminology that not only summarizes our interpretations of morphological homology and discusses the most analysed characters in the genus *Scaptomyza*, but also can be adopted for future descriptions in this genus.

**Table 1.** Preferred terms for head sclerites and the synonyms adopted in the literature

Preferred term	Synonyms	Figure	References
Vertex	Vertex	5	[12–14,16,28,29,33–36,40,49,79,108,109,113,114]
	Epicranium		[28]
Frons	Frons		[9,13,14,18,28,30,31,33–40,43,45,46,48,50,58,61,63,67,70–72,75,76,79,98–100,108,109,112–114]
	Front		[10,16,17,28,41,42,47,49,51–54,57,59,60,62,64–66,68,78,97,104,108,109,114]
	Frontal region		[69]
	Postfrons		[28]
Fronto-orbital plates	Fronto-orbital plates	5	[18,28,29,31,32,73–75,98,100,108,109,113,114]
	Frontal orbital plates		[30]
	Frontal orbits		[44,54,99]
	Orbitae		[48,50]
	Orbital plates		[9,33]
	Orbits		[13,16,17,28,34–43,45,46,49,52,54,58,63,65,78,79,97,114]
	Parafrontal plates		[28,108,109]
	Periorbits		[47,51,53,57,59–62,64,71]
Ocellar triangle	Ocellar triangle	5	[9,13,17,18,28–33,41–43,45–49,51–54,57–65,67,68,70–76,79,97,98,100,108,109,114]
	Ocellar region		[34]
	Space between the ocelli		[78]
Frontal triangle	Frontal triangle	5	[9,13,18,32,33,37,50,77,98,99]
	Long triangle nearly reaching the forehead		[14]
	Triangle		[30,35–37,39,45]
	Vertical triangle		[78]
Frontal vitta	Frontal vitta	5	[28,33,73,74,108,109,113]
	Interfrons		[28,108,109]
	Interfrontal area		[108,109]
	Mesofrons		[28,108,109,113]
Ptilinal fissure		5	[28,108,109]
	Ptilinal suture		[33,74]
Face	Face	2	[9,10,12–14,16,17,28–31,33–46,48–50,52–54,57–67,69–71,73–76,78,96,98–100,108,109,112–114]
	Prefrons		[28]
Facial carina	Facial carina	2, 3	[18,28,29,34,108,109,112,113]
	Carina		[16,17,30,31,35–43,45–54,57–64,66–74,79,96–99,104]
	Keel		[10,14,17,44,78]
Eye	Eye	2,3	[9–11,13,14,16–18,29–45,47–53,57–61,63–76,79,96–98,100,104,112,114]
	Compound eye		[28,108,109,113]
Occiput	Occiput	3	[16,17,28–30,33,34,48,49,52,53,57,59,66–68,73,74,77–79,97,98,108,109,113,114]
	Occipital areas		[54]
	Postcranium		[28]
Gena	Gena	3	[16–18,28,31,33,35–38,40,50,52,73,74,99,108,109,113,114]
	Bucca		[28]
	Cheek		[13,14,28–30,34,41–43,45,47,49,51,53,54,57–64,66,68,70–72,76,78,97,98,104]
	Jowl		[28,44,46]
Clypeus	Clypeus	2	[16,17,28–30,33,42,44,46–48,51–54,57–61,63,64,69–71,73–76,100,108,109,113,114]
	Anteclypeus		[28]
	Clypeal margin		[41]
	Frontoclypeus		[108,109]
	Prelabrum		[28,40]
Proboscis	Proboscis	2	[9,10,13,16,18,28–30,32,33,41,42,44,49,57,62,66,67,69,77,78,108,109,113]
Palpus	Palpus	2,3	[9–11,14,16–18,28,30–42,44–54,57–65,67–74,76–79,98–100,108,109,113,114]
	Maxillary palpus		[66,108,109]
	Palp		[29,108,109]
Labellum	Labellum	2,3	[29,30,33,108,109,114]
	Labella		[17,28,52,113]

**Table 2.** Preferred terms for head setae and the synonyms adopted in the literature

Preferred term	Synonyms	Figure	References
Postocellar	Postocellar	4	[9,17,18,28,29,31–33,108,109,112–114]
	Postvertical		[13,14,16,17,35–40,42,49,54,57,58,60,61,63,67,68,79,97–99]
Four verticals			[99]
	Post-orbitals		[14]
	Verticals		[36,38,44,46,50,68,97]
Inner vertical	Inner vertical	4	[13,14,16–18,28–32,35,37,39,40,42,45,48,50,54,61,63,67,69,74,98,100,108,109,112]
	Internal vertical		[63]
	Medial vertical		[9,33,108,109,113]
Outer vertical	Outer vertical	4	[13,14,16–18,28–30,35,37,39,40,54,69,108,109,112]
	Lateral vertical		[33,108,109,113]
Ocellar setae	Ocellar setae	4	[9,13,14,16,17,28–31,33,35–38,40,52,54,58,61–63,68,73,74,97,99,108,109,112–114]
Proclinate	Proclinate	4	[16,17,31,34,36,37,41,45,49,52,53,56,59–62,64,65,67,69,70,100,114]
	Anterior fronto-orbital		[43]
	Anterior orbital		[18,32,98]
	Anterior proclinate		[13]
	First orbital		[51,63]
	Or 1		[58]
	Orb 1		[9,33]
	Orb 3		[57]
	Proclinate orbital		[28,30,35,38–40,42,54,66,68,71,74,79,97,99,112]
	Third orbital		[16]
	Upper proclinate		[63]
Anterior reclinate	Anterior reclinate	4	[17,30,31,36,37,41,49,52,54,60,62,69,70,75,100,114]
	Anterior reclinate orbital		[29,35,39,42,45,46,53,56,59,61,64–66,68,71,97]
	Lower orbital		[28,112]
	Lower reclinate		[17,34,38,40,79,114]
	Median reclinate		[13]
	Mid orbital		[18,32,98]
	Middle fronto-orbital		[43]
	Middle orbital		[16,41,66,67]
	Or 2		[58]
	Orb 2		[9,33,57]
	Second orbital		[16,51,63]
	Second reclinate		[63]
	Small reclinate		[35]
Posterior reclinate	Posterior reclinate	4	[13,18,30,31,41,48,49,59–62,64,67,69,70,98]
	First orbital		[16]
	Or 3		[58]
	Orb 1		[57]
	Orb 3		[9,33]
	Posterior fronto-orbital		[43]
	Posterior orbital		[32,44]
	Posterior reclinate orbital		[29,42,45,46,53,56,65,68,71,74]
	Third orbital		[63]
	Third reclinate		[63]
	Upper orbital		[16]
	Upper orbital		[28,112]
	Upper reclinate		[17,34,37,38,40,44,79]
	Upper reclinate orbitals		[35,46,99]
Interfrontal setulae	Interfrontal setulae		[29,33,73,74,98,108,109,113]
	Hairs on the anterior margin of the interfrontalia		[99]
Vibrissa	Vibrissa	2,3	[9,13,14,16,18,28–33,35,37,38,40,42,46–50,53,59–61,64,65,68–71,73,74,76,78,79,99,108,109,112,113]
	First oral		[16,18,33,41,42,45,54,56,66,72,97]
	First vibrissa		[62]
	One strong oral		[67]
	Oral bristle		[58,63]
	Oral vibrissa		[100]
	Prominent oral bristle		[51]
	Uppermost bristle of the vibrissal row		[17]
Subvibrissal	Subvibrissal	2,3	[28–30,108,109,113]
	Buccal bristles		[50]
	Other orals		[61]
	Peristomal		[108,109]
	Second vibrissa		[62]
	Second oral		[16,18,33,41,42,45,48,49,53,54,56,59,60,63–66,70–72,97]
	Slightly shortly bristles adjacent to the vibrissa		[35]
	Subvibrissa		[69,74]

**Table 3.** Preferred terms for antennae characters and the synonyms adopted in the literature

Preferred term	Synonyms	Figure	References
Scape	Scape	6	[28,33,108,109,113]
Pedicel	Pedicel	6	[28–31,33,73,98,108,109,113,114]
	Second antennal joint		[11–14,42,46,47,50,54,58–60,64,66,71,78]
	Second antennal section		[42]
	Second antennal segment		[16,17,35,49,53,57,63,68,72,75,76,97,100,114]
First flagellomere	First flagellomere	6	[9,18,28,29,31–33,73,74,108,109,113,114]
	Basal flagellomere		[108,109]
	Flagellomere I		[28–30,69,98,108]
	Postpedicel		[108,109]
	Third antennal joint		[10–14,41,45–47,50,54,59–62,64,66,71,78]
	Third antennal section		[58]
	Third antennal segment		[16,17,34,35,37,38,40,42,45,49,53,54,57,58,62,63,68,70,72,75,79,97,99,100,108,109,114]
Dorsal branches	Dorsal branches	6	[9,16,29,30,33,41,42,46,48,50,53,54,58–61,64,66,67,69,70,72,74,76,97]
	Branches above		[16,57,62,104]
	Dorsal hairs		[63]
	Dorsal rays		[13,17,52,55,75,96,100,112,114]
	Hairs above		[39,78]
	Hairs on upper side		[10]
	Rays above		[31,35,38,40,68,97,99]
	Rays above fork		[47]
	Upper branches		[18,32,39,58,71,98]
	Upper rays		[14,35,38,45]
Ventral branches	Ventral branches	6	[9,16,29,30,33,42,46,48,50,51,53,54,58–61,64,66,67,69,70,72,74,76,97]
	Branches below		[16,41,49,57,62,65,104]
	Hairs below		[39,78]
	Lower branches		[18,32,39,58,71,74,98]
	Lower hair		[63]
	Lower rays		[14,35,38,45]
	Rays below		[31,35,36,40,68,97,99]
	Rays below fork		[47]
	Ventral rays		[13,17,52,55,96,100,112,114]
Inner branches	Inner branches		[9,18,32,33]
	Central branches		[48]
	Inconspicuous setae along inner margin		[114]
	Inner row		[14]
	Minute medial branches		[30,69]
	Short hairs on the inner surface of the arista		[17]
	Short, hair-like branches on its inner side		[16]
Terminal fork	Terminal fork	6	[9,18,30–33,41,42,45,46,49,53,57–59,62,63,66,68,70–72,75,76,97,100,104]
	Apical fork		[17,52,54,55,58,67,114]
	Bifurcate apically		[112]
	Distal fork		[67]
	End fork		[48,50,96]
	End ray		[14]
	Fork		[47,51,59,64,65]
	Small fork		[60,61]
	Terminal bifurcated		[57]
	Terminal bifurcation		[29,73,74]

**Table 4.** Preferred terms for thorax sclerites and the synonyms adopted in the literature

Preferred term	Synonyms	Figure	References
Scutum	Scutum	7	[9,18,28,32,33,73,74,98,108,109,112,113]
	Mesonotum		[13,16,17,31,34–43,45–48,50–63,65–68,70,72,75,76,96,97,99,100,108,109,114]
	Mesoscutum		[64]
	Thoracic dorsum		[79]
	Thorax		[12,77,78]
Scutellum	Scutellum	7	[9,12–14,16–18,28–35,37,38,40–43,45–50,52–55,57–78,97,99,100,108,109,112–114]
Pleura	Pleura		[9,13,16–18,29–31,33–35,37,40–43,45–47,50,52–54,57–64,66–68,70,71,75,77–79,100,114]
	Pleural region		[69]
	Pleuron		[28,114]
Postpronotum	Postpronotum	9	[28,31,33,112]
	Humeral callus		[41]
	Humerus		[16]
Notopleuron	Notopleuron	9	[33,108,109]
	Propleura		[16,52]
Anepisternum	Anepisternum	9	[28,31,33,100,108,109,112,113]
	Mesopleura		[16,37,38,40,42,52,58,62]
	Mesopleuron		[17,68,73,74,108,109]
Anepimeron	Anepimeron	9	[28,31,33,108,109,112,113]
	Metasternum		[58]
	Pteropleura		[16,42,52,59]
	Pteropleuron		[68,108,109]
Katepisternum	Katepisternum	9	[28,33,100,108,109,112]
	Mesosternum		[13]
	Sternopleura		[16,37,39,42,62]
	Sternopleuron		[43,58,108,109]
Meron	Meron	9	[28,108,109]
	Hypopleura		[16,17,42]
	Hypopleuron		[108,109]

**Table 5.** Preferred terms for dorsal thoracic setae and the synonyms adopted in the literature

Preferred term	Synonyms	Figure	References
Presutural dorsocentral	Presutural dorsocentral		[9,17,31,36,40,42,44,48–50,58,75,96,100]
	Dorsocentral bristles anterior to the suture		[98]
Dorsocentrals	Dorsocentrals	7	[9,13,14,16,28,31,34,39,48,63,66,67,75,98,100,104,108,109,113,114]
	Postsutural dorsocentrals		[96]
Anterior dorsocentral	Anterior dorsocentral	7	[17,18,29,30,32,33,35–38,40–42,45,47,49,50,52,53,57–62,64,66,68–72,74,76,97,99,104,112]
	Median dorsocentrals		[42]
Posterior dorsocentral	Posterior dorsocentral	7	[17,18,29,30,32,33,35–37,40–42,47,50,52–54,57–62,64,68–72,74,76,97,99,112]
Supra-alars	Supra-alars	7	[14,16,17,28,29,35,104,108,109,112,113]
	Prealars		[35]
	Presutural supra-alar		[74]
Anterior supra-alar	Anterior supra-alar	7	[17,33]
	First pair of supra-alars		[52]
	First supra-alar		[17]
Posterior supra-alar	Posterior supra-alar	7	[33]
Post-alars	Post-alars	7	[14,16,28,35,99,108,109,112,114]
Anterior postalar	Anterior postalar	7	[33]
Superior postalar	Superior postalar	7	[33]
Acrostichals	Acrostichals	7	[9,14,16–18,28–33,36,38,41,42,44–46,48–76,96–100,104,108,109,112–114]
	Intradorsocentral acrostichals		[45]
	Intradorsocentrals		[35–40,79]
Prescutellars	Prescutellars		[9,13,14,16–18,30–33,41,42,45,49,53,57,58,62,64,65,67–69,71,74,113]
	Prescutellar acrostichals		[31,35,37,40,79,99,112]
Scutellars	Scutellars	7	[36,55]
Apical scutellars	Apical scutellars	7	[18,28,29,31–35,40–42,44,46,48–50,54,63,66,69,74,104,108,109,112,113]
	Apical marginal bristles		[13]
	On their extreme tips (of the scutellum) are the crossed terminal pair		[14]
	Posterior marginals		[43,58]
	Posterior scutellars		[45,47,48,53,57–64,68,70–72,75,76,97,100]
	Upper scutellars		[17]
Basal scutellars	Basal scutellars	7	[17,18,28,31–35,40–42,46,48–50,63,73,74,108,109,112,113]
	(Scutellum) bears basally two long bristles		[14]
	Anterior marginals		[58]
	Anterior scutellars		[29,41,43,45,47,48,53,57–64,66–72,75,76,97,100,114]
	Lateral scutellars		[44]

**Table 6.** Preferred terms for lateral thoracic setae and the synonyms adopted in the literature

Preferred term	Synonyms	Figure	References
Postpronotals	Postpronotals	8	[28,73,108,109,113]
	Humerals		[14,16,17,29,34–39,42–45,47,49–51,53,57,62,63,65–67,69,75,96,99,100,108,109,114]
Anterior postpronotal	Anterior postpronotal	8	[108,109]
	Upper humeral		[41,46,48,52,54,58–61,64,71]
	Upper posterior humeral		[79]
	Upper postpronotal		[18,33]
Basal postpronotal	Basal postpronotal	8	[108,109]
	Lower humeral		[41,46,48,52,54,58–61,64,71]
	Lower postpronotal		[18,33]
Notopleurals	Notopleurals	8	[13,14,16,28,29,33,35,42,69,108,109,112]
Posterior notopleural	Posterior notopleural	8	[17,99]
Presutural bristle	Presutural bristle	8	[16]
Katepisternals	Katepisternals	8	[28,29,31,69,108,109,112,113]
	Sternopleurals		[14,35–40,43,49,50,62,63,79,96,108,109]
Anterior katepisternal	Anterior katepisternal	8	[9,18,32,33,74,75,98,100,114]
	Anterior sternopleural		[17,41,42,48,52,58,97]
Middle katepisternal	Middle katepisternal	8	[33]
	Median katepisternal		[9]
	Mid katepisternal		[18,32,98]
	Middle sternopleural		[41,58,97]
Posterior katepisternal	Posterior katepisternal	8	[9,33,74,75,100,114]
	Posterior sternopleural		[17,41,42,52]

**Table 7.** Preferred terms for leg characters and the synonyms adopted in the literature

Preferred term	Synonyms	Figure	References
Coxa	Coxa	10	[14,16–18,28,29,33,37,41,42,48,49,51–54,57,62,66,69,75,77,78,98,100,103,108,109,113,114]
Trochanter	Trochanter	10	[14,16,17,28,33,37,44,48,49,53,54,58,66,69,74,75,100,103,108,109,113,114]
Femur	Femur	10	[13,14,16–18,28–31,33–49,52–55,57–60,62,63,65,66,69,74,75,78,79,98–100,103,108,109,112–114]
Tibia	Tibia	10	[9,13,14,16–18,28,29,31–42,44,45,47–49,51–55,57–64,67–76,78,79,97,99,100,102–104,108,109,112–114]
Preapical (tibial) seta	Preapical (tibial) seta	11	[9,13,16,33,40–42,45,47,49,51–53,57,59–61,63,64,67,68,70–72,76,97,104,108,109,113]
	Dorsal preapical		[32]
	Preapical dorsal		[35,37,38,69,73,74,112]
	Preapical dorsal spine		[17]
	Preapical spur		[113]
	Subapical		[31,58]
	Subapical dorsal		[74]
Apical (tibial) seta	Apical (tibial) seta	11	[9,16,33,58,68,71–73,76,97,104,112]
	Apical spur		[31,67]
	Terminal spur		[14]
	Ventral apical		[32]
Tarsus	Tarsus		[12,14,17,18,28,29,31,33–40,42,48,49,51–54,57,59,61–63,69,75,77,78,99,100,108,109,113,114]
Tarsomeres	Tarsomeres	10	[33,108,109,113]
	Tarsal joints		[16,18,41,47,59,60,64,65,71]
	Tarsal segments		[70,103,104]
Claw	Claw	12	[11,14,16,28,35,49,108,109,113]
	Tarsal claws		[37,108,109]
	Toes		[57]
	Unguis		[108,109]
Pulvilli	Pulvilli		[28,53,108,109,113]

**Table 8.** Preferred terms for wing and halteres characters and the synonyms adopted in the literature

Preferred term	Synonyms	Figure	References
Wings	Wings	13	[9–14,16–18,28–54,57–79,97–100,102,104,108,109,112–114]
Halter	Halter	14	[9,12–14,16–18,28–42,45–51,53,54,57–66,68–74,76,79,98,99,108,109,113]
	Balancer		[16]
Halter knob	Halter knob	14	[9,17,28,30,31,33,34,36–38,49,53,54,58,68,70,108,109]
	Tip		[62]
Halter stem	Halter stem	14	[28,31,108,109]
	Stalk		[9,33,57,68,70]
Halter base	Halter base	14	[28,30,54,58,62,108,109]

**Table 9.** Preferred terms for male terminalia characters and the synonyms adopted in the literature

Preferred term	Synonyms	Figure	References
Epandrium	Epandrium	17	[9,18,28–33,58,63,64,67,70–74,76,98,100–103,105–112]
	Dorsal sclerite		[108–110]
	Genital arch		[15,16,28,41,46–49,51,53,54,56,57,59–61,64–68,95,97,102,104,112]
	Ninth tergite		[61,100]
	Ninth tergum		[17,52,55]
	Periandrum		[108–110]
	Tergite 9		[108–111]
Cercus	Cercus	17	[9,18,28–33,44,46,50,52,61,64,70–74,76,98,100,101,105–110,112]
	Anal cerci		[48]
	Anal lamellae		[44]
	Anal plates		[15–17,42,45–47,49,51–56,58–61,63–65,67,68,76,95,97,102,104]
	Cercal plate		[103]
	Rudiments of segment 11		[28]
Cercal ventral lobe	Cercal ventral lobe	18	[101]
	Cercal clasper		[103]
	Narrower anterior lobe of the clasper		[17]
	Paralobe		[46,48,50,53,59,66,76,98]
	Posterior clasper		[16]
	Secondary anal plate		[95]
	Secondary clasper		[31,50,54,56,57,66,76,95–97,104]
	Secondary forceps		[58]
	Subsurstylus		[96]
	Ventral cercal lobe		[33]
Subepandrial sclerite	Subepandrial sclerite		[101,105–110]
	Bacilliform sclerite		[28,105–110]
	Decasternum		[9,18,30,32,33,47,53,104,108–110]
	Mediandrium		[108–110]
	Processus longi		[110]
	Sternite 10		[28,108–110,112]
	Tenth sternite		[74]
	Ventral epandrial plate		[28,112]
	Ventral epandrial sclerite		[108,109]
Surstylus	Surstylus	20	[9,18,28–33,64,70–76,96,98,100–102,105–110,112]
	Clasper		[16,17,38,49,51–53,55,56,59–61,64,65,67,68,102,104]
	Forceps		[35,46,48,50,63,67]
	Primary clasper		[15,47,54,57,66,95–97,104]
	Surstylar clasper		[103]
Surstylar teeth	Surstylar teeth	20	[101]
	Denticles		[96]
	Peg-like bristles		[16]
	Prensisetae		[9,29,30,32,33,73,74,76,108–110,112]
	Primary teeth		[67,104]
	Spines		[42]
	Teeth		[15,17,18,31,46–49,51–53,56,57,59–61,63–65,68,70–72,95,97,98,102,103,112]
Hypandrium	Hypandrium	21, 22	[9,17,18,28–33,46,50,55–61,63,64,67,68,74,75,98,100,101,105–112]
	Hypandrial lobe		[73]
	Hypandrial plate		[53]
	Ninth sternum		[17,52]
	Novasternum		[47,51,65,70,72,76]
	Sternite 9		[108–111]
	Ventral sclerite		[108–110]
Gonocoxite	Gonocoxite	21, 22	[101,105–111]
	Basimere		[108,109]
	Basistylle		[108–110]
	Coxite		[108,109]
	Gonocoxa		[108,109]
	Gonopod	21, 22	[9,18,28–30,32,33,74,98,112]
Pregonite	Pregonite	23, 24	[101,106–110]
	Anterior gonapophysis		[54,56,57,70,76]
	Anterior paramere		[31,47,51–53,59–61,64,65,71–73,104]
	Outer paraphysis		[33]
Postgonite	Postgonite	23, 24	[101,106,108–110]
	Posterior gonapophysis		[76]
	Posterior paramere		[47,52,71,72,97,104]
	Inner paraphysis		[33]
Phallus	Phallus	23, 24	[58,101,105–110]
	Aedeagus		[9,17,18,28–33,46,47,51–53,55,59–62,64,65,68,70–74,97,98,100,101,104–112]
	Male copulatory apparatus		[57]
	Penis		[35,56,57,108–110]
	Phallosome		[108–110]
Phallapodeme	Phallapodeme	23, 24	[101,106–110]
	Aedeagal apodeme		[9,18,28–33,74,98,105,107–110,112]
	Apodeme		[60,64]
	Apodeme of aedeagus		[59,61]
	Basal apodeme		[53,55,70]
	Basal apodeme of aedeagus		[72]
	Ejaculatory apodeme		[105–110]
	Penis apodeme		[46]

**Table 10.** Preferred terms for female terminalia characters and the synonyms adopted in the literature

Preferred term	Synonyms	Figure	References
Epigynium	Epigynium	25	[28,108,109]
	Tergite 8		[28,33,108,109]
Epiproct	Epiproct	25	[18,28,30,33,108,109,112]
	Long-haired dorsal chitinized plate		[16]
	Supra-anal plate		[108,109]
	Tergite 10		[108,109]
Hypoproct	Hypoproct	25, 26	[18,28,30,33,108,109,112]
	Long-haired ventral chitinized plate		[16]
	Subanal plate		[112]
Hypogynial valve	Hypogynial valve	25, 26	[28,108,109]
	Egg guides		[47,51,53,57,60–62,64,65,68,70,72,96,97,104]
	Egg-guide lobes		[59]
	Ovicauda		[28]
	Oviposition tube		[28]
	Ovipositor		[14,28,31,42,45,46,54,58,61,63,66,71,75,78,100,108,109,114]
	Ovipositor blades		[17,52,55]
	Ovipositor guides		[48,50,67]
	Ovipositor plates		[16–18,41,44,98,112]
	Oviscape		[29,69,108,109]
	Oviscapt		[9,28,30,32,33,74,76]
	Shining serrated plates		[10]
Ovisensilla	Ovisensilla	25, 26	[9,29,30,32,33,69,74,114]
	Coarse teeth		[45]
	Denticles		[48]
	Discal teeth		[47]
	Longer marginal bristles		[14]
	Marginal dentation		[96]
	Ovisensillum		[76]
	Peg-like bristles		[16]
	Spines		[44,63]
	Teeth		[17,18,42,46,51–53,57–61,64–66,68,70–72,98]

**Figures 2, 3.**
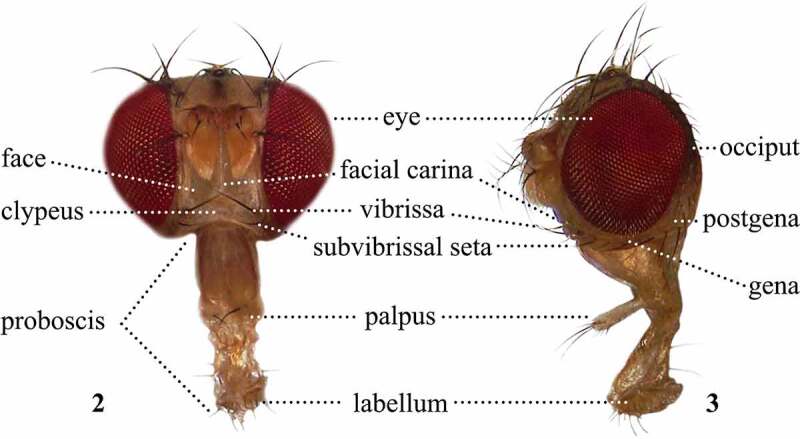
Frontal (2) and left lateral (3) views of the sclerites and setae of the head and proboscis of *Scaptomyza pallida.*

**Figures 4, 5.**
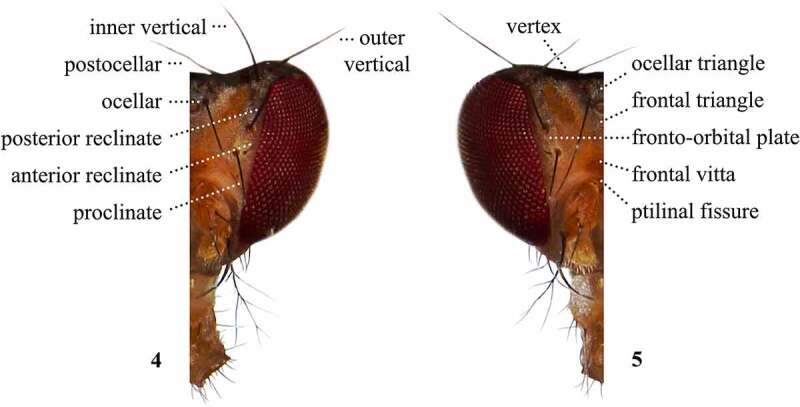
Setae of the head, right side, dorsal view (4); Sclerites of the head, left side, dorsal view (5) of *Scaptomyza pallida.*

**Figure 6.**
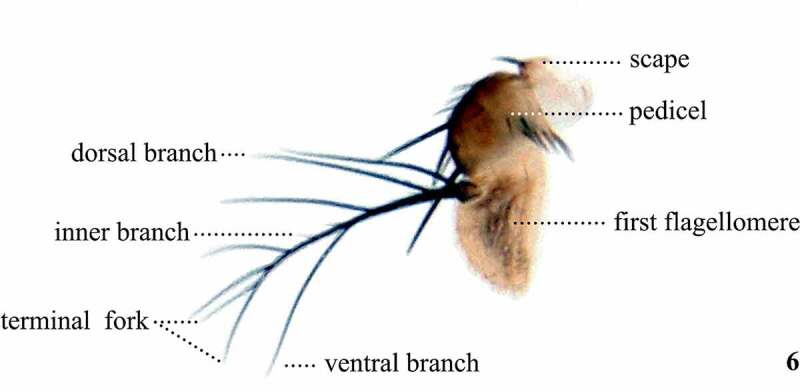
Antenna of *Scaptomyza pallida.*

**Figure 7.**
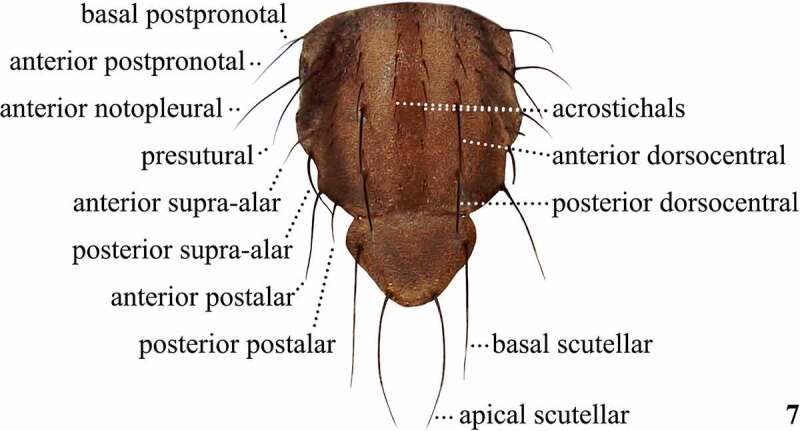
Setae of the thorax of *Scaptomyza pallida*, in dorsal view

**Figures 8, 9.**
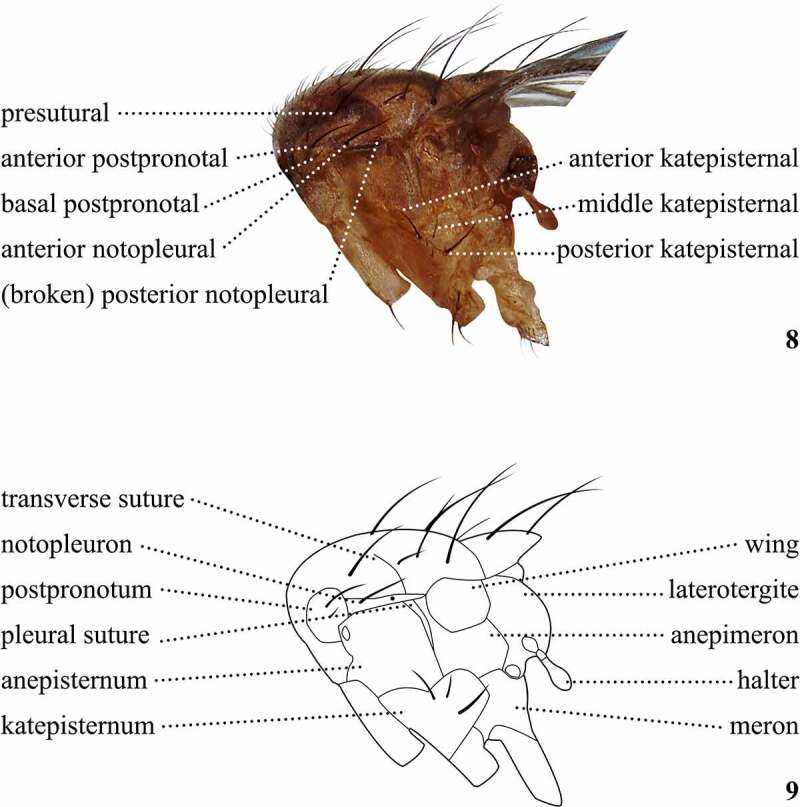
Setae of the thorax, left lateral view (7); Contour of the thoracic sclerites, left lateral view (8) of *Scaptomyza pallida.*

**Figures 10-12.**
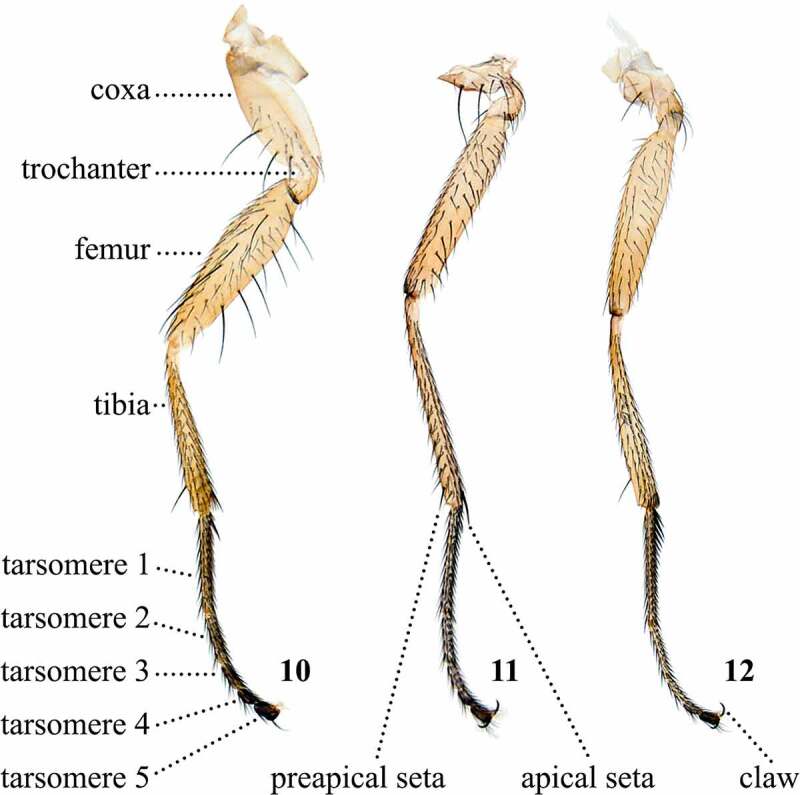
Fore leg (10), mid leg (11), and hind leg (12) of *Scaptomyza pallida.*

**Figure 13.**
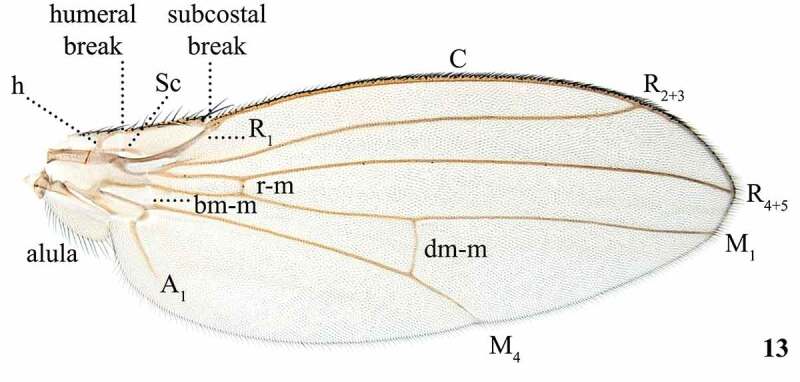
Wing of *Scaptomyza pallida*. Abbreviations: h = humeral; Sc = subcosta; C = costa; R_1_, R_2+3_, R_4+5_ = radial veins; M_1_, M_4_ = medial veins; A_1_ = anal vein; r-m, bm-m, dm-m = crossveins

**Figure 14.**
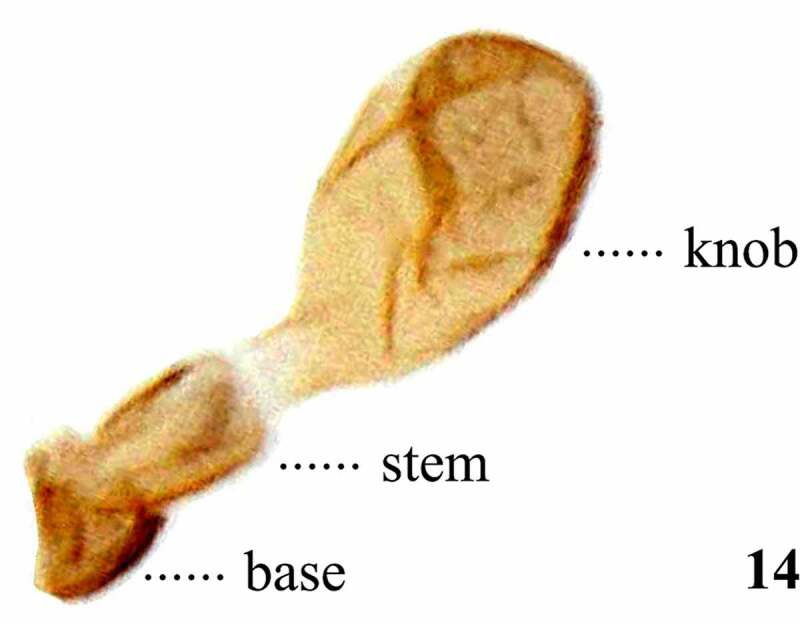
Left lateral view of the halter of *Scaptomyza pallida.*

**Figures 15, 16.**
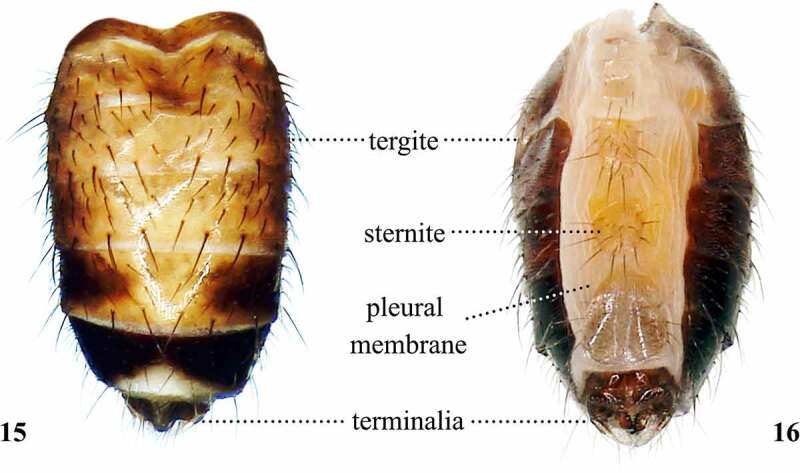
Dorsal (15) and ventral (16) views of the abdomen of *Scaptomyza pallida.*

**Figures 17-20.**
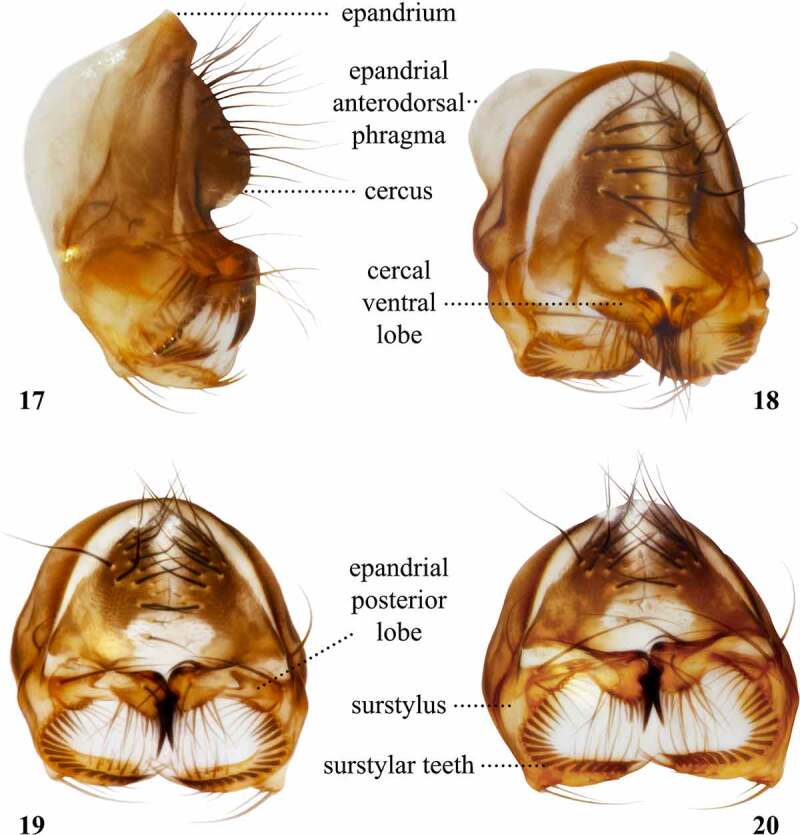
Left lateral (17), oblique posterior (18), posterior (19), and posteroventral (20) views of the epandrium and associated sclerites of the male terminalia of *Scaptomyza pallida.*

**Figures 21-24.**
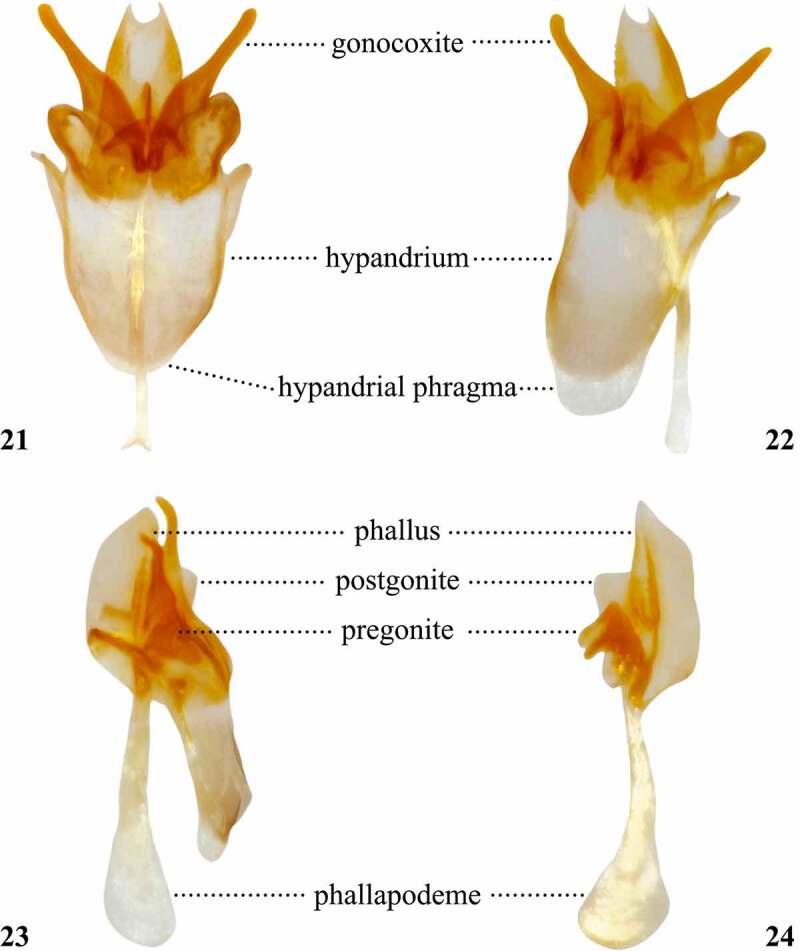
Posterior (21), oblique posterior (22), left lateral (23) views of the hypandrium and associated sclerites; right lateral (24) view of the phallus, postgonites, pregonites, and phallapodeme of the male terminalia of *Scaptomyza pallida.*

A number of terms refer to characters present on multiple body regions, such as chaetotaxy or colouration. The following terms were adopted, after McEvey [31], with previously used synonyms in parenthesis: setae (bristles or spines), setulae (hairs), and stripes (vittae). It is worth defining two terms commonly used in species descriptions: pollinosity and chaetotaxy. Pollinosity (pruinescence) refers to a pigmentation pattern overlaying the ground cuticle colour, which often resembles fine dust or coarse powder. Chaetotaxy can either refer setae and setulae on any part of the exoskeleton or to their general position, orientation, and arrangement [28,120]. We created a visual atlas of *Scaptomyza pallida* to provide a clear link between the terms (Tables 1–Tables 10) and the observed morphology (Figures 2–26). We focus our discussions on variable characters commonly used in species descriptions in order to reduce ambiguity and inaccuracy in future taxonomic work. Those characters that are invariant or not applicable to species descriptions are not treated.

**Figures 25-26.**
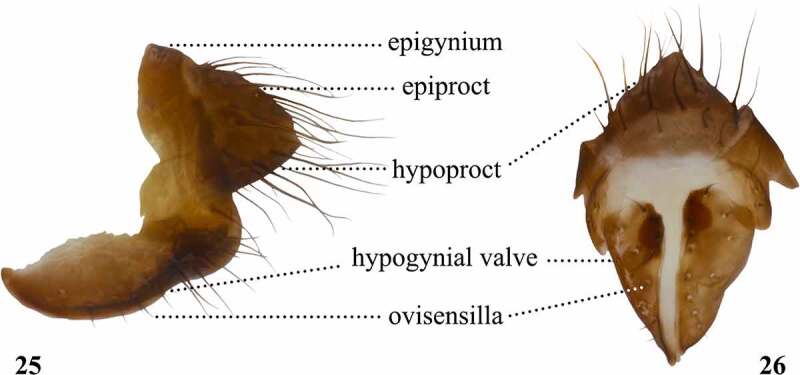
Left lateral (25) and posterior (26) views of the female terminalia of *Scaptomyza pallida.*

## Head

The most often described head sclerites are the ocellar triangle, fronto-orbital plates, frontal vitta, facial carina, and gena (Figures 2–5). Species descriptions often include their colourations and relative lengths [17,33]. We adopt the term ‘frons’ to refer to the entire region between the eyes, the vertex and the ptilinal fissure, and bearing the ocellar triangle (Figure 4–Figure 5). The lateral region of the frons corresponds to the fronto-orbital plates, whereas the central region corresponds to the frontal vitta, which may bear the interfrontal setulae on its distal portion [28,29,33,113]. The term ‘frontal vitta’ refers to the sclerite itself (Figure 5) [120]. Various taxa bear pigmented stripes in this area that may extend over other head sclerites. We recommend that when describing the presence of colour patterns, authors to be specific about which sclerites bear these patterns. The term ‘keel’ has been used with two different connotations in the literature. Some authors have adopted it as a synonym to facial carina [10,14,44,78], whereas it has also been used as a modifier to describe a facial carina that is distinct and narrow at the tip [17]. To avoid ambiguity, we recommend authors to use the term keel to describe the facial carina’s shape and not as a synonym.

Head setae (Figures 2–4) are also often analysed when describing species. Authors frequently include the arrangement, relative position, and size of orbital setae, as well as the number and relative size of oral setae [17,33]. The large, compound eyes of drosophilids have been traditionally referred to simply as eyes (Figure 2–3). Their size, shape, and colour are important diagnostic characters [16,17,33]. The mouthparts (Figure 2–3) may also be included on descriptions, usually focusing on the colour of the palpi and proboscis [17,33]. Early authors used vague terms while referring to mouthparts, including ‘sucker’[11] and ‘tongue’[14]. We interpret these authors were referring to the labellum and associated structures, such as the pseudotrachea, *sensu* McAlpine *et al* [28].

It is worth noting that some authors refer to the two pairs of vertical setae (Figure 4) without distinction [99], whereas we follow McAlpine *et al* [28]. and refer to each pair individually, as inner and outer vertical setae. Another author [17] used the expression ‘vertical and upper ocellar bristles’ when erecting the subgenus *Grimshawomyia*. Species belonging to this subgenus have an extra pair of head bristles, which are inserted near the inner and outer verticals, in a swollen region at the proximal portion of the fronto-orbital plates. In this publication, we refer to the three setae located at the fronto-orbital plates following McEvey [31], naming them proclinate, anterior reclinate, and posterior reclinate setae (Figure 4). They are usually collectively named orbital setae [16], but had also been called fronto-orbital setae [14].

Throughout *Scaptomyza* descriptions, there are terms used to refer to multiple head sclerites collectively that have not been widely adopted by modern taxonomists and systematists. The parafrontalia [114] includes the region ranging from frontal vitta, along ptilinal fissure, face, and facial carina. The term interfrontalia [37,38,50,99] refers to the sclerites in the frontal part of the head, while interorbitalia and interorbital area [50] refer to the region between the orbital plates. The epistome [13,35–39,63,99] and epistoma [17,58,63,114] refer to the lower facial margin, but these terms are ambiguous and should be avoided [28,120,121]. Finally, the peristoma [10,58] refers to sclerites surrounding the proboscis, such as the clypeus and gena [120]. We include these here for clarity but do not endorse their use and, instead, suggest that authors specify individual sclerites when preparing descriptions.

## Antennae

The antennae (Figure 6) are divided into 3 segments, the scape, pedicel, and first flagellomere [28]. Attached to the first flagellomere is the arista, and the presence and number of dorsal and ventral branches (rays), as well as how deep the terminal fork is, are important characters to define *Scaptomyza* species and subgenera [17,75]. In addition, the shapes of the first flagellomere and pedicel are also diagnostic characters [17,75] and their colour is frequently included in species descriptions [9]. Walker [11] used the term ‘feelers’ to refer to the antennae and Okada [44] used the expression ‘hairs in front of arista’ to refer to the setulae on the first flagellomere. These terms are non-specific and should be avoided in modern species descriptions.

## Thorax

Early publications used the terms thorax and scutellum in descriptions [12,77,78] when referring to the dorsal surface of the thorax. Therefore, the term thorax was probably referring exclusively to the scutum. Later, Grimaldi [29,30,69] used the term ‘notum’ to refer to the whole dorsal surface of the thorax, including the pronotum, mesonotum, and the postnotum, although this has not been followed by other drosophilists. The term mesonotum comprises essentially the entire dorsal surface of the mesothorax in Diptera and is divided into prescutum, scutum, scutellum, and postnotum [28,108,109]. The scutum is by far the largest portion of the mesonotum, located between the pronotum and the scutellum, and is divided into a presutural area and a postsutural area by the transverse suture [28,108,109]. It is worth noting that in the past the term mesonotum have been misapplied to the scutum alone [28,108,109]. One of the most conspicuous thoracic characters (Figures 7–9) in the genus *Scaptomyza* is the colouration pattern and presence of stripes on the dorsal region of the scutum and scutellum (Figure 7).

The lateral region of the thorax (Figure 8–9) is complex and contains many individual sclerites, the chaetotaxy and colouration of which may also be important in diagnosing species [17,33]. Various authors have used a number of terms to refer to subdivisions of this region, although these are generally confusing and not uniformly applied. For example, several authors [16,42,58,77,78] have used ‘metanotum’ to refer to the region that bears the halter and the posterior spiracle, being delimited by the anepimeron, meron, and the abdomen [16]. However, in other Diptera, this region is called laterotergite, and sometimes is divided into two sclerites, a dorsal anatergite and a ventral katatergite [28]. There is no suture dividing the laterotergite in drosophilids, which suggests these sclerites may have been fused or one of them is extremely reduced or invaginated following the divergence of this family. Likewise, Malloch [37] used ‘prosternum’ for the region adjacent to the first coxa and ventral to the humerus, comprising the preapisternum, preapimeron and anepisternum (*sensu* McAlpine *et al* [28].). Here, we propose that workers refer to specific sclerites by name and generally avoid ambiguous terms referring to multiple sclerites. The exception to this recommendation is the term pleuron (*pl*. pleura), which includes the anepisternum, anepimeron, and katepisternum [33] and is the most frequently analysed sclerites among Drosophilidae taxonomists and systematists.

The relative position and size of thoracic setae, including the postpronotals, katepisternals, dorsocentrals, and scutellars (Figures 7–8) are taxonomically important in Drosophilidae [17,33]. Many *Scaptomyza* species, as with most Drosophilidae, have two pairs of postsutural dorsocentral setae (Figure 7), referred to as anterior and posterior dorsocentrals [17]. However, all species belonging to the subgenus *Rosenwaldia* have an extra pair, located at the presutural portion of the scutum [17,48,75]. In this case, we will follow Hardy [17] and name them presutural, anterior, and posterior dorsocentral setae, respectively. The orientation of the scutellar setae (Figure 7) is also important, and they may be divergent, parallel, or convergent [33]. The number of acrostichal setae rows (series) between the anterior pair of dorsocentral setae (Figure 7) is used to define not only *Scaptomyza* subgenera but also the whole genus [10,17,75].

## Wings

The use of the term ‘wing’ to refer to the first membranous pair of wings (Figure 13) and ‘halter’ to refer to the second, modified pair (Figure 14) is constant throughout the Drosophilidae literature [16,17,33]. Some variation in nomenclature is observed when referring to different regions of the halters, which often possess distinctive colouration used in descriptions [17,28,33,62]. We divide halters into three sections, the knob, stem, and base, according to McAlpine *et al* [28]. We follow the wing venation nomenclature of Cumming & Wood [108], which adopts the alternative wing venation system based on clearer homologies between Diptera and Mecoptera fore wing base, proposed by Wootton & Ennos [122] and Saigusa [123], instead of the traditional system used in McAlpine [124] and Merz & Haenni [113]. Even though most of the nomenclature of Drosophilidae wing venation is consistent between these two systems, the following different terms were proposed, with the traditional terminology presented parenthetically: M_1_ (M), M_4_ (CuA_1_), bm-m (bm-cu), and dm-m (dm-cu).

## Legs

The major leg divisions (Figure 10–12), *i.e*. the coxa, trochanter, femur, tibia, and tarsus [16,17,33], are universally adopted throughout *Scaptomyza* literature. The tarsus is subdivided into five segments, which have been referred to as tarsomeres [33,113], tarsal segments [70], or tarsal joints [16]. At the tip of the tarsomere 5 there are the claws. We believe the term tarsal joint (*sensu* Sturtevant [16]) is ambiguous, since ‘joint’ usually refers to the connection between segments, and not to the segments themselves. Therefore, we discourage its use in favour or more specific language elaborating characters present on each segment. Colour patterns on the legs, as well as presence of tibial setae and size of tarsomeres relative to one another are important diagnostic characters in *Scaptomyza* [17].

## Abdomen

When the abdomen (Figure 15–16) is included in descriptions, authors usually focus on the size, overall shape, colour, and presence of bands or dots on the abdominal tergites [17,33]. The most important abdominal characteristics are located at its tip, called terminalia.

## Male terminalia

Early researchers [16] referred to the male terminalia as the hypopygium. As this character became more commonly used to diagnose drosophilid species, the level of detail increased the need of a more specific nomenclature for the complex structures of the male terminalia. Researchers developed techniques to finely dissect the terminalia [15,33,55,115,125], which would then be drawn under a compound microscope attached to a *camera lucida*, allowing drosophilists to prepare detailed fine line drawings [45]. As imaging technology progressed, it became possible to obtain photomicrographs attaching cameras into compound microscopes [126]. Later, it became possible to assess the terminalia morphology without the need of dissecting, using scanning electron microscopy [75]. Currently, the state-of-the-art technology that may be used to get high-resolution imaging of the terminalia is the micro computed tomography scan [127], which enables virtual dissection of the sclerites.

We follow the updated nomenclature proposed by Rice *et al* [101]., which comprehensively revised the terminology adopted to refer to male terminalia sclerites of *Drosophila melanogaster*. In the model organism *Drosophila melanogaster*, the intromittent organ is named phallus, and comprises the aedeagus, postgonites and aedeagal sheath [101]. The revised epandrial hypothesis proposes an interpretation of homologous male terminalia in the Eremoneura [107], in which the aedeagus and the aedeagal sheath (parameral sheath *sensu* Cumming *et al* [107].) are fused to form a composite structure termed the phallus [128], observed in the Stratiomyomorpha and Muscomorpha (*sensu* Woodley [129]). Accordingly, the interpretation in Bächli *et al* [33]. also consider that the aedeagus has been fused to the aedeagal sheath (referred to as inner paraphysis), forming a more or less sclerotized structure referred to as aedeagus in most derived Drosophilidae, including species belonging to the subgenus *Drosophila*. Interestingly, it is possible to find an intermediate state in the *Lordiphosa denticeps* species group, in which the aedeagus is partially fused to the aedeagal sheath [130].

The level of detail used in describing male terminalia varies according to the technique used. Without dissecting, it is possible to observe the epandrium, cerci, surstyli, and the tip of the aedeagus, provided it is protruded. This is particularly true if modern technology has been used, such as scanning electron microscopy [75]. However, dissections allow researchers to observe the morphology of other sclerites that are indistinct or located internally. By separating the terminalia from the abdomen, it becomes possible to observe the overall shape of the hypandrium and part of the phallapodeme [17]. Disarticulating all sclerites makes it possible to clearly observe the morphology of the subepandrial sclerite, hypandrium, gonocoxites, pregonites, postgonites, phallus, and phallapodeme in great detail [9,33] (Figures 17–24).

When describing *Scaptomyza* species, authors often focus on the sclerites that are visible after dissecting the male terminalia from the abdomen, but without disarticulating sclerites from each other. The most conspicuous characters analysed are the width, presence of setae, and modifications on epandrium, cerci, surstyli, and the ventral margin of the hypandrium, named ventral fragma [47,51,53,59,61,70,72,76] or hypandrial phragma [101]. If the phallus is protruded, its length and overall shape, especially of the tip, have also been included [17,48,52,75,96] (Figures 17–24). When describing the male terminalia and its sclerites it is often useful to examine them from three axes: left-right axis, antero-posterior axis, and dorso-ventral axis.

The ventral lobes of the cerci of some species of Drosophilidae resemble the surstyli typically found on the epandrial sclerite (Figure 17–20). Sometimes these modified structures also bear modified setae that are similar to, yet stronger than, surstylar teeth [54]. Authors have referred to the cercal ventral lobes [101] as ventral cercal lobes [33], paralobes [89] or secondary claspers [54]. Interestingly, the modified cercal ventral lobes have evolved multiple times in the Drosophilidae, including the genus *Scaptomyza* and the *melanogaster* species group of the genus *Drosophila* [104]. The epandrial sclerites are also heavily modified in some *Scaptomyza* species and may include long and narrow epandrial ventral or posterior lobes, projected alongside the surstyli, which have been referred to as tertiary clasper or toe [54] (Figure 17–20). Other significant modifications used to define species are the lateral lobes on the dorsal region of the hypandrium in the subgenus *Grimshawomyia*, which are projected beyond the surstyli; as well as one pair of very well developed gonocoxites, conspicuously visible even without dissecting in the subgenus *Alloscaptomyza*.

## Female terminalia

Cumming & Wood [108] recently provided homology statements for structures of the female terminalia within the order Diptera and we will follow their terminology. These are similar to those proposed by McQueen *et al* [131]. in their comprehensive revision of female structures in *Drosophila melanogaster*. While Cumming & Wood [108] did not specifically address chaetotaxy of the female terminalia, Bächli *et al* [33]. did and we will adopt their naming conventions in the current paper. The female terminalia is not as variable as the male terminalia within the genus *Scaptomyza*. Even though these organs are not suitable for species delimitation, there are variable hypogynial valves across Hawaiian subgenera. For instance, females belonging to the subgenera *Alloscaptomyza, Bunostoma, Elmomyza, Engiscaptomyza, Grimshawomyia, Parascaptomyza, Rosenwaldia*, and *Tantalia* have weakly sclerotized, fleshy hypogynial valves that does not bear ovisensilla. On the other hand, species belonging to the *Exalloscaptomyza* subgenus have sclerotized hypogynial valves, fused on both ends, whereas *Titanochaeta* females have unusually sclerotized, needle-like, and pointed hypogynial valves, which may be correlated with their ecology, since their larvae use spider egg sacs as their breeding sites [17,75].

The most important characters in species descriptions are the shape of the hypogynial valves, number and overall shape of ovisensilla, and eventual presence of other setae [17,33] (Figure 25–26). In addition, some authors also use internal characters to define species, such as spermatheca, ventral receptacles, and parovaria [57,58,67,96,104,125,132]. Although not usually included in descriptions, it is worth noting the epiproct and hypoproct [18,28,30,33,112] have also been referred to as long-haired anal cerci [44] and anal plates [17,52]. The term ‘basal isthmus [60,104]’ refers to the anteroventral connection between the hypogynial valves. The meaning of the term ‘genital lamellae of female’[35] remains unclear and will require additional research.

## Conclusions

The terminology revision and the visual atlas provided in the present study should facilitate the interpretation of historical *Scaptomyza* descriptions, linking the older literature with modern terminology. In addition, we proposed a standardized terminology for future descriptions, which will be adopted in upcoming revisions of Hawaiian *Scaptomyza* subgenera. Additional studies comparing the male terminalia morphology between the *Scaptomyza* subgenera, as well as across other genera within the family Drosophilidae with similar terminalia modifications will help us better understand the evolution of this remarkably modified character.

## Data Availability

The authors confirm that the data supporting the findings of this study are available within the article.
